# Understanding reasons for discontinued antiretroviral treatment among clients in test and treat: a qualitative study in Swaziland

**DOI:** 10.1002/jia2.25120

**Published:** 2018-07-19

**Authors:** Fortunate S Shabalala, Eva Vernooij, Christopher Pell, Njabulo Simelane, Nelisiwe Masilela, Donna Spiegelman, Boyang Chai, Shaukat Khan, Ria Reis

**Affiliations:** ^1^ Department of Anthropology Amsterdam Institute for Social Science Research University of Amsterdam Amsterdam the Netherlands; ^2^ Department of Community Health Nursing Sciences Faculty of Health Sciences University of Swaziland Mbabane Swaziland; ^3^ Department of Social Anthropology School of Social and Political Science University of Edinburgh Edinburgh UK; ^4^ Amsterdam Institute for Global Health and Development Amsterdam the Netherlands; ^5^ Harvard T.H. Chan School of Public Health Boston MA USA; ^6^ Clinton Health Access Initiative (CHAI) Mbabane Swaziland; ^7^ Department of Public Health and Primary Care Leiden University Medical Centre Leiden the Netherlands; ^8^ The Children's Institute School of Child and Adolescent Health University of Cape Town Cape Town South Africa

**Keywords:** ARV, Retention, Linkage to care, Intervention, Swaziland, Test and Treat

## Abstract

**Introduction:**

Retention on antiretroviral therapy (ART) is critical for the successful adoption of the test and treat policy by sub‐Saharan African countries, and for realizing the United Nations programme on HIV and AIDS target of 90‐90‐90. This qualitative study explores HIV positive clients’ reasons for discontinuing ART under the *Max* ART test and treat implementation study in Swaziland.

**Methods:**

Clients identified as lost to follow‐up (LTFU) in the programme database, who had initiated ART under the intervention arm of the *Max* ART study, were purposively selected from two facilities. LTFU was defined as stopping ART refill for three months or longer from the date of last appointment, and not being classified as transferred out or deceased. Semi‐structured face‐to‐face interviews were conducted with nine clients and one treatment supporter between July and August 2017. All interviews were conducted in the local language, audio‐recorded, summarized or transcribed and translated to English for thematic analysis.

**Results:**

Respondents described mobility as the first step in a chain of events that affected retention in care. It was entwined with precarious employment, care delivery, interactions with health workers, lack of social support, anticipated stigma and ART‐related side‐effects, including the exacerbation of hunger. The chains of events involved several intersecting reasons that occurred one after the other as a series of contiguous and linked events that led to clients’ eventual discontinuation of ART. The individual accounts of step‐by‐step decision‐making revealed the influence of multi‐layered contexts and the importance of critical life‐events.

**Conclusions:**

Clients’ reasons for abandoning ART are a complex, inextricably interwoven chain of events rather than a single occurrence. Mobility is often the first step in the process and commonly results from precarious economic and social circumstances. Currently the health system poorly caters to the reality of people's mobile lives. Interventions should seek to increase healthcare workers’ understanding of the chain of events leading up to discontinuation on ART and the social dilemmas that clients face.

## 1 Introduction

Between 2000 and 2015, the number of people living with HIV (PLHIV) who access antiretroviral therapy (ART) saw a thirty‐fold global increase: from 250,000 to more than 17 million 1, 2. With increasing evidence of the benefit of “early” ART initiation in reducing morbidity, mortality, and onward transmission of HIV 3, 4, 5, the World Health Organization's (WHO) 2015 consolidated treatment guidelines recommended “test and treat”: ART for all people diagnosed with HIV, regardless of CD4 cell count or disease stage.

With countries across sub‐Saharan Africa (SSA) adopting test and treat, there is a renewed need to ensure long‐term retention on ART. Across Africa, the retention of clients in HIV care remains a challenge, with continent‐wide retention estimated to be 65% at 36 months 6. The high attrition rates mainly result from loss to follow‐up 7, 8, 9. This is of concern because interrupting or discontinuing ART leads to suboptimal clinical outcomes, higher risks of opportunistic complications, loss of income, and death 10, 11. Reasons for loss to follow‐up include a lack of food, religious and family influences, use of traditional or alternative medicine, enacted and/or perceived stigma, poor client‐provider relationships, improvement in health status and treatment fatigue 8, 11, 12. Stemming high loss to follow‐up rates is key to sustaining gains made against the HIV epidemic.

In Swaziland, with a reported prevalence of 30.5% among 18‐49 year‐olds, HIV is the leading public health concern 13. Although HIV incidence reduced from 2.5% in 2011 to 1.4% in 2016, prevalence has remained stable 13, 14. By December 2016, 171,266 (of the estimated 220,000) Swazi PLHIV had initiated ART, representing a coverage of 78% 15. Among PLHIV in Swaziland, the 2016 retention at 36 months was 85% 15, indicating relatively high retention in care. Little is known, however, about the reasons for disengagement from care among the remaining 15%.

In general, the population of Swaziland is extremely mobile, within and across borders, and this has been a key driver of the HIV epidemic 16. In 2010, almost half of 946 surveyed Swazi clients identified as lost to follow‐up (LTFU) could not be traced because of their high mobility 17. It is crucial to understand how mobility – absence from one's place of residence for a prolonged period of time – is linked to other reasons for discontinuing ART to design effective interventions to improve retention in care under test and treat. Drawing on interviews with clients identified as lost to follow‐up in two public health facilities, this article explores their reasons for discontinuing ART. Ultimately, the aim is to use these data to inform an assessment tool to proactively identify clients likely to discontinue ART and offer them stepped‐up counselling and support.

## 2 Methods

### 2.1 Study setting

Data were collected as part of the social science component of *Max*ART, a multidisciplinary implementation study in Hhohho Region, Swaziland, which examined feasibility, acceptability, affordability, and scalability of test and treat 18. The MaxART study protocol is described in detail elsewhere 18. The *Max*ART study population included all consenting adult (above 18 years) PLHIV and ART‐naive clients. Pregnant and breastfeeding mothers and/or unable/refusing to consent were excluded. The *Max*ART clinical database included all study participants and was frequently updated with data from the 14 participating public health facilities. The data presented herein were collected at a regional referral hospital and a rural clinic, purposively selected because they were high‐volume sites and reported the highest number of LTFU clients in the *Max*ART database.

Ethical approval was obtained from the Swaziland National Health Research Review Board.

### 2.2 Data collection

In the *Max*ART database, clients who stopped ART refill for three months or longer from the date of last appointment and were not registered as either deceased or transferred out from the two sub‐study sites were classified as LTFU. All LTFU clients from the selected sites were contacted using their mobile phone numbers obtained from the *Max*ART database. The calling researcher explained the purpose of the study to potential participants over the phone and verified whether they had difficulties with taking ART. Potential respondents who confirmed they had stopped treatment were asked whether they were willing to be interviewed about their experiences. An appointment was set with those who agreed and a day before the agreed date respondents were called again to confirm their availability. Before the interview took place, the study purpose was explained again to the participants and written or verbal informed consent was obtained according to the participant's preference. Anonymity and confidentiality were maintained throughout the study.

Clients who were not reachable through their mobile phone number were traced through their listed treatment supporters. For those who could still not be reached, the treatment supporter was interviewed if he or she was aware of the client's HIV status. Among those successfully traced, eleven clients were confirmed as LTFU. All eleven clients (three males and eight females), and one treatment supporter were approached for an interview. One client refused, another evaded the researchers, not keeping four consecutive appointments. In one case, although the client could not be traced, her treatment supporter was interviewed. The findings are therefore based on face‐to‐face interviews with nine LTFU clients and one treatment supporter. Using a semi‐structured topic guide, trained and experienced social scientists carried out the interviews that explored their reasons for discontinuing ART.

Consistent with an inductive approach, interviews were designed to be flexible, following a general, topic‐oriented structure. The interview guide contained open questions on reasons for and experiences with discontinuation of ART, HIV testing, meaning of positive results, disclosure, availability (or lack) of a support system at home, the health facility and the community, and intentions to restart ART in the future. All interviews were conducted in the local language (siSwati) and audio‐recorded upon consent from clients. Most audio recordings were transcribed verbatim and as data started to become saturated, and no new themes emerging, the final audio recordings were summarized. All transcripts/summaries were translated to English for analysis.

### 2.3 Data analysis

In consultation with co‐authors, FS manually analysed the data, using a data‐driven inductive thematic approach to steer cross‐case comparison. According to Thomas 19, the general inductive approach “allows research findings to emerge from the frequent, dominant, or significant themes inherent in raw data collected, without the restraints imposed by structured methodologies.” A narrative approach was also used to elicit in‐depth understanding of the individual contexts in which decisions were made. This was combined with constant comparison as described by Glaser and Strauss 20. Combining cross‐case analysis and within‐case analysis helps to maintain the contextual richness of individual experience 21. During analysis, emerging patterns were shared and discussed with EV and RR. Initial findings were discussed with all co‐authors.

## 3 Results

### 3.1 Respondent characteristics

All clients enrolled in *Max*ART, who initiated ART between January 2014 and October 2016 and were classified as LTFU were eligible for inclusion. From the two selected facilities, 145 clients were identified as LTFU: 81% (n = 118) were aged 25 to 39 years, 58% (n = 84) were female, and 57% (n = 83) initiated ART with CD4 < 350 cells/mL. Of the 145 clients, 93 (64%) could not be reached when called because their mobile numbers were either not available, unknown to the person answering or the call was unanswered. Clients (or the treatment supporters of those that could not be reached) were called five times on average.

Fifty‐two clients (36%) were contacted by phone. Of these, 26 were actively attending the facility but records had not been updated, 14 had transferred out from the (“mother”) facility where the client initiated ART to a new one, and one person had died. During the call, eleven clients (three males and eight females) confirmed having discontinued ART (Table 1).

**Table 1:** Outcome of contact tracing of the initial LTFU selection

LTFU client categories	Total (N=145)
Unable to contact	93 (64%)
Contacted	52 (36%)
Confirmed LTFU^a^	11 (21%)
Active on ART^b^	26 (50%)
(Self)Transferred Out	14 (27%)
Died	1 (2%)

a Clients who had stopped ART for 90 or more days from the last clinic appointment date

b Clients who reported that they were still on ART at the same facility where they were reported as LFTU

### 3.2 The chain of events leading to discontinued ART

Initially most respondents reported mobility – relocating residence – as the main reason for discontinuing ART. Further probing revealed more complex circumstances: sub‐optimal care from health care providers, severity and prolonged medication side effects, fear of stigma, lack of food and social networks were entwined in the process that leads to loss to follow‐up. In‐depth analysis of individual cases revealed several intersecting reasons that occurred consecutively, as a “chain of events” 22. Thus, a series of contiguous and linked events led to their eventual discontinuation of ART. The individual accounts of step‐by‐step decision‐making revealed the influence of multi‐layered contexts and the importance of critical life‐events.

### 3.3 Mobility as the first step to discontinuation of treatment

For over half of the respondents, the first step in the chain of events towards stopping ART was relocation to another town or community far from the health facility where HIV treatment was obtained. Life events, such as caring for a loved‐one residing in another area or changes in employment, often prompted relocation.

I went to Mankayane to look after my brother who was sick… I took the pills with me and left my card thinking that I would not stay long. But the pills were finished while I was still there. (Female client, LTFU 05).

I had just started working after a long time without a job. Continuing with the pills would have meant I had to ask for a day off every month to go to the clinic. I have children to take care of… My husband died four years ago so I am their [children] only provider. I feared that if I continued with the pills my employer would fire me… I couldn't risk that… Also I quit treatment because I tested when I was still staying at home which is at [name of residential area]. When I found a job in Mbabane, it became too far for me to fetch the tablets at [name of health facility]. (Female client, LTFU 03).

As the previous quote illustrates, the mobility that leads to loss to follow up is related to precarious social and economic living circumstances which led some respondents to choose (continuation of) employment over continuation of treatment. Economic motives were prominent in respondents’ explanation of why they stayed away from their place of residence for prolonged periods of time. In some cases, the distance to the mother facility, or a clash in working hours at the facility and those of respondents’ jobs made it difficult for clients to continue to access ART. Jobs involving travel, such as driving a taxi or employment far from home made accessing care more difficult. Often, however, mobility alone did not necessarily threaten continued treatment; rather it became a problem in the context of the organization of HIV care in the public health system.

### 3.4 The health system response to mobility

An immediate consequence of mobility was that clients had to (re)gain access to care. Returning after temporary absence meant regaining access to one's previous clinic. Negative experiences with healthcare providers sometimes became intertwined in the process that led to discontinuing ART. A participant who relocated because of her brother's sickness describes:

I went to the nurses at the ART clinic with the pill container to ask them to at least give me a few pills and explained my situation. But they refused and told me they needed the card to dispense the pills and write on it. I even asked one of the lodgers to go with me to plead with the nurses but they refused. Because I did not have money to go to [the facility where she gets ART] I stopped. After my brother's discharge from hospital I went back to my clinic, but they reprimanded me for skipping the pills for three weeks…The lady who wears maroon told me I was not serious and I am wasting their time. She took me to the counsellor who also shouted at me… So I told myself I will not go back there. (Female client, LTFU 05).

Perceived lack of attention or empathy from healthcare providers came to the fore in several accounts. These respondents mentioned that they decided to stop ART after they could not get the assistance they sought. Respondents reported being spoken to “roughly”, “shouted at” or feeling that the clinic staff “didn't care”. Such harsh treatment left clients feeling hurt, angry, and humiliated. One such client abandoned care due to poor care for her child by the HCWs, when she sought their assistance:

My child had problems but they did not want to listen, instead they shouted at me. (Female client, LTFU 09).

Others did not like that they had to “retell their story” to new HCW:

I have thought about going there [nearest facility in the new residence] but eish, you know I thought now I have to retell my story again. So I thought, eish, I will see as time goes on…That was six months ago. (Female client, LTFU, 09).

For some respondents, the experience of side‐effects became intertwined in the process of stopping treatment. As part of initiating ART, clients were informed about the possibility of side effects and assured that they will resolve over a few days or weeks. However, the experience of severe side effects coupled with a perceived lack of attention or empathy from HCWs resulted in some respondents stopping ART:

I was tired of taking [the tablets] because they also distorted my body shape. It became bad, I started to develop a hump at the back, and my belly was big… [HCWs] told me that the pills were the cause of that… I reported that the pills were giving me problems but they did not do anything about it. They kept telling me it will be better with time but it didn't. It's like they just didn't listen or care about me… So I stopped. I just stopped the pills. (Female client, LTFU 01).

The complex processes resulting in clients abandoning ART also influenced their decisions to re‐engage in HIV care. Fear of reprimand by HCWs was a prime reason for respondents’ reluctance to re‐engage in care.

### 3.5 Economic and social circumstances underlying mobility

Uncertain economic circumstances and unemployment led respondents to seek jobs elsewhere and to food insecurity, which was compounded by the feelings of hunger respondents associated with ART:

You know my sister, these pills are very good but, ey, they demand that you eat a lot. Because I am not employed and do not have money to buy food I decided to stop taking them. Maybe I will go back when I get a job and are able to buy food. (Male client, LTFU 07).

Others mentioned that ART made them fall ill, which meant lost income or unemployment:

When I started taking the pills I experienced abdominal pains and diarrhoea. They also caused bile… I was very sick and bedridden… I left my workplace to go home because of the sickness caused by the pills… I decided to stop them… I feel ok now [after stopping the ARVs], so I see no reason for returning to start the treatment again, especially because they made me very sick when I took them. (Male client, LTFU 10).

Mobility also disrupted the stability of or accessibility to support networks. A treatment supporter described how the influence of her granddaughter's peers led to her running away from home and abandoning treatment. For one respondent, whose job required him to spend weeks away from home, being abandoned by his wife, who acted as his treatment supporter, led to him stopping HIV treatment:

When she left I had no one [to fetch the ARVs]… Okay, I missed my appointment but the tablets were still there. Then the tablets got finished and as time went by I just thought that there was nothing to do. Then I said let the will of God be done, if I die then I'll die… I reached a time that I gave up since I felt I am no longer a person in my family. They don't like me. I think if I can die maybe. I think I have two or three weeks still deliberating about this… So I am nothing at home so that is why I thought if only it was possible for God to take me, let him take me because I can't commit suicide since it is a sin… (Male client, LTFU 08)

For this man, the negative psychological impact of family troubles became intertwined in the chain of events that led to disengagement from care. But his story also highlights how harmonious social relations (e.g. a supportive partner) may mitigate or compensate for mobility related challenges to continuing antiretroviral treatment.

### 3.6 Negative emotions as a cross‐cutting theme

Psychological factors were mentioned by most participants, with negative emotions (e.g. anxiety, fear) shaping decisions about care. As mentioned, nurses’ lack of understanding about the circumstances that caused clients’ mobility and complicated adherence led to feelings of hurt and anger. Anticipated stigma also featured prominently: concerns about stigma made respondents reluctant to disclose their HIV‐status and they sometimes felt continuing treatment could lead to unintentional disclosure. Anxieties about unintended disclosure were related to the risk of partner violence or abandonment, losing a job, or social marginalization and, for some, became an added reason for discontinuing ART. This was particularly the case for women who are economically and socially dependant on male partners:

I found a partner and I couldn't bring myself to tell him. I did not tell him in the beginning [when we met] so it became hard to continue taking the tablets because he would find out… So I thought what if he became violent, or leave me, something like that. So I decided not to tell him. (Female client, LTFU 01).

For some respondents, a positive health outcome combined with fear of stigma became the final reason to discontinue ART. Having lied to their partners about the pills they were taking while visibly sick, made it difficult to continue taking them when they looked well again. Rather than risking being “caught” by her partner, one respondent opted to stop taking treatment:

My partner could see that I was now alright. So, if I continued taking the pills how could I explain that? So I stopped… He will leave me if I tell him [about my HIV status]. (Female client, LTFU 06).

Mobility figured as a primary trigger in some narratives about loss to follow‐up, but ultimately it was respondents’ navigation of the precarious social, economic *and* medical landscape that led to disengagement from care. In this chain of events, decisions were shaped by practical reasoning and emotional appraisals.

## 4 Discussion

The interviews with LTFU clients in Swaziland reveal how mobility can trigger a chain of events that leads to disengagement from care. In the process that leads to loss to follow‐up, mobility is often entwined with precarious employment, care delivery, interactions with health workers, lack of social support, anticipated stigma and ART‐related side‐effects, including the exacerbation of hunger.

Reasons for discontinuing ART have been typically described as complex 22, 23. The thematic analysis revealed similar reasons for disengagement from care to those described by Ware et al. who identified competing social and economic demands, violence, lack of family or community support, and dissatisfaction with care 22. Several studies have identified HIV‐related stigma as a barrier to accessing ART or retention in care 24, 25. Fear of violence and/or rejection by a partner is commonly reported as a barrier to accessing HIV care, particularly for women. Extensive research highlights the relationship between HIV status and intimate partner violence following disclosure by women 26, 27, 28, including in Swaziland 25, 29. In a similar way to stigma, violence can be anticipated or enacted. Across SSA, most women who disclose their HIV status report supportive reactions from their partners, whereas a few experience partner violence and abandonment 30. Similarly, in this study, no female respondents described violence; rather they feared that intentional or accidental disclosure of their HIV status would lead to violence. Other studies have also described fear of losing one's job because of HIV 31, 32.

The narrative analysis revealed how a chain of events had triggered step‐by‐step their decisions to discontinue treatment. In this process, reasons became intertwined in complex and individualized ways. Respondents initially described relocation or mobility as their main reason, but closer scrutiny of their accounts shows that mobility resulted from complex individual navigations of precarious and specific life circumstances and was often triggered by critical life‐events. The death of one's spouse, a brother's serious illness, the marriage break‐up of a migrant labourer, or the lack of freedom to refuse mobile employment or a job far from home, are experienced and presented as highly individualized events pertinent to the micro scale of everyday life.

In Swaziland, these events typify the insecure living conditions for the majority and are intrinsically linked with macro‐level processes. Respondents who explained their financial predicament and the difficult choices about balancing their responsibility to their own health and caring for others, are victims of a precarious economy affected by the HIV epidemic.

Health policies that steer the procedures for patient transfers from one clinic to another are meso‐level structural processes that do not align with the mobile lives of Swazis. Respondents’ negative appraisals of their treatment by health staff, and the need to retell their illness trajectories over and over again speak of health providers’ frustrations and challenges with structural health system issues – specimen transportation and additional administrative tasks of test and treat – beyond their control. This also pertains to the difficulties of healthcare staff to respond to clients’ need for food and medication without the side‐effects that undermine their quality of life.

In resource‐limited, high‐HIV‐prevalence settings, the influence of contextually embedded everyday practices and structures means that engagement with HIV services must be considered in relation to other social practices 23. Ultimately, for Swazi clients, chains of events that complicate such engagement take shape in a context of dynamic and emotionally charged relationships with partners, families, peers and colleagues. Fear of stigma, violence and/or being rejected, and psychological distress related to loss can be the proverbial straw that breaks the camel's back.

The findings suggest that policy interventions to prevent loss to follow‐up should be comprehensive, multifaceted, and address the organization of healthcare as well as be tailored to the situations and needs of individual clients. At health system level, policy makers and implementation scientists should pay attention to how referral systems and inter‐facility communications can be improved to support healthcare workers to provide care to mobile clients who need temporarily access to HIV care in different localities. Interventions to prevent disengagement from care must consider the varied, complex and processual nature of factors involved in individuals’ care trajectories, and recognize the key issues around re‐engaging with care.

Because such trajectories are largely unconnected to clients’ specific characteristics, but are rather linked to specific occurrences in their lives, healthcare providers must recognize such events as risks to disengagement from care, understand how they accumulate, and be able to effectively intervene. Healthcare providers need (knowledge, attitude and skills) training to help clients overcome difficulties to staying in treatment or to facilitate re‐engagement in care of those who temporarily abandon treatment. Health staff must be informed of the social and economic challenges that LTFU clients face, plus their reasons for stopping ART. They also need an in‐depth understanding of clients’ efforts to re‐engage with care. Training is also needed to strengthen healthcare workers’ ability to reflect on their feelings (of disappointment, failure, anger, prejudice) when faced with clients who (temporarily) abandon treatment, to avoid these (understandable) feelings becoming a factor in the process leading to clients discontinuing treatment.

Finally, healthcare workers must be trained to identify signs of disengagement from care and to intervene at any point in the chain of events to help clients re‐engage with care. A concise and practical (decision‐making) tool that would help staff to attend more closely to clients’ dilemmas and needs and identify solutions is needed. Mobility, as a potential starting point for a chain of events that leads to loss to follow‐up, should be a focus in this instrument.

### 4.1 Strengths and limitations

This is the first study in Swaziland to explore – from clients’ perspectives – reasons for discontinuation of ART under test and treat in a context of high mobility. Combining in‐depth thematic and narrative analysis highlighted the complex sequential intertwinements of reasons for disengagement. Using qualitative methods, this study revealed the importance of social and psychological ramifications of critical life‐events and structural issues often overlooked in quantitative studies of decision‐making. More interviews are needed to establish typical first events and chains of events that lead to disengagement from care. The data reflects the perspectives of clients who had disengaged from care and additional interviews with HCWs, family members, and employers would have been beneficial. A systematic comparison with the narratives from people confronted by similar events and circumstances while continuing treatment would foster understanding of resilience to disengagement from care. Another limitation is that respondents were enrolled in a large intervention study with quality and ethics of care potentially superior to the norm in other facilities.

## 5 Conclusions

In Swaziland, there have been remarkable achievements in terms of the rates of HIV testing and ART initiation. Disengagement from HIV care however threatens this success. Swazi clients’ reasons for abandoning ART are a complex, inextricably interwoven chain of events rather than a single occurrence. During these processes, clients take action to navigate the challenges they face before deciding to stop ART. Mobility – temporary or permanent relocation far from the health facility where HIV treatment was obtained – is often the first event in such a process. Mobility commonly results from complex deliberations weighing economic, social and other circumstances, and becomes a problem because the health system poorly caters to the reality of people's mobile lives.

## Competing interests

The authors declare that they have no competing interests.

## Authors’ contributions

EV and RR conceived the sub‐study; FS and SK designed the sub‐study protocol; NS and NM conducted the interviews under the supervision of FS and SK; FS conducted data analysis in consultation with SK, EV and RR. FS, EV and RR drafted the manuscript; CL, DS, BC and SK critically revised the manuscript for intellectual content. All authors read and approved the final manuscript.

## Funding

This work, as part of the *Max*ART Consortium, was supported by the Dutch Postcode Lottery in the Netherlands, the Embassy of the Kingdom of the Netherlands in South Africa/Mozambique, British Columbia Centre of Excellence in HIV/AIDS, Mylan and Médecins Sans Frontières.
